# Membrane-Free Lateral Flow Assay with the Active Control of Fluid Transport for Ultrasensitive Cardiac Biomarker Detection

**DOI:** 10.1021/acs.analchem.4c00142

**Published:** 2024-04-25

**Authors:** Dan Strohmaier-Nguyen, Carina Horn, Antje J. Baeumner

**Affiliations:** † Institute of Analytical Chemistry, Chemo- and Biosensors, University of Regensburg, 93053 Regensburg, Germany; ‡ Roche Diagnostics GmbH, 68305 Mannheim, Germany

## Abstract

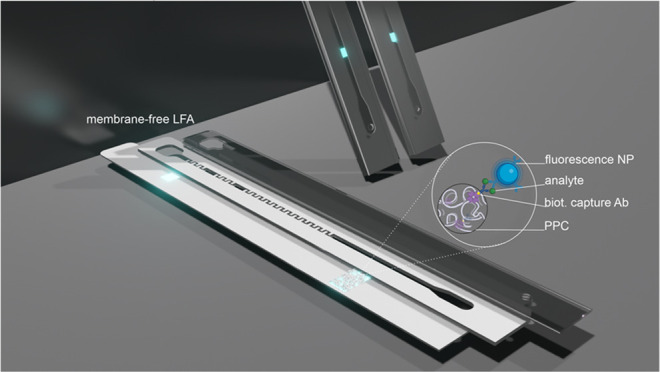

Membrane-based lateral flow immunoassays (LFAs) have been employed as early point-of-care (POC) testing tools in clinical settings. However, the varying membrane properties, uncontrollable sample transport in LFAs, visual readout, and required large sample volumes have been major limiting factors in realizing needed sensitivity and desirable precise quantification. Addressing these challenges, we designed a membrane-free system in which the desirable three-dimensional (3D) structure of the detection zone is imitated and used a small pump for fluid flow and fluorescence as readout, all the while maintaining a one-step assay protocol. A hydrogel-like protein–polyelectrolyte complex (PPC) within a polyelectrolyte multilayer (PEM) was developed as the test line by complexing polystreptavidin (pSA) with poly(diallyldimethylammonium chloride) (PDDA), which in turn was layered with poly(acrylic acid) (PAA) resulting in a superior 3D streptavidin-rich test line. Since the remainder of the microchannel remains material-free, good flow control is achieved, and with the total volume of 20 μL, 7.5-fold smaller sample volumes can be used in comparison to conventional LFAs. High sensitivity with desirable reproducibility and a 20 min total assay time were achieved for the detection of NT-proBNP in plasma with a dynamic range of 60–9000 pg·mL^–1^ and a limit of detection of 56 pg·mL^–1^ using probe antibody-modified fluorescence nanoparticles. While instrument-free visual detection is no longer possible, the developed lateral flow channel platform has the potential to dramatically expand the LFA applicability, as it overcomes the limitations of membrane-based immunoassays, ultimately improving the accuracy and reducing the sample volume so that finger-prick analyses can easily be done in a one-step assay for analytes present at very low concentrations.

Recently, with the continuous process in point-of-care (POC) testing, fast and ultrasensitive detection is accomplished by lateral flow immunoassays (LFAs).^1,2^ The simple, affordable, and user-friendly setup makes the LFA a relevant and efficient diagnostic tool where high-tech infrastructure may not be possible. The fundamental part of the LFA is the porous membrane that enables passive sample migration through capillary forces and straightforward immobilization of proteins necessary for detection such as antibodies and streptavidin.^3,4^ However, the LFA also bears some major drawbacks and limitations such as variations in flow rate and analysis time due to varying pore structure and sample viscosity, obstruction of pores by matrix components, reasonably high sample volume due to its inherent absorbing properties, and inconsistency in the dispersion of the labeled sample to the membrane due to batch-to-batch variations of the membrane.^5,6^ In addition, as the LFA performance strongly depends on the properties of the membrane material, where a change of the production line by the supplier results in changes in the membrane properties due to modifications of production parameters such as drying temperature and line speed, requiring reoptimization of an already finalized assay. In addition, Mosley et al. demonstrated that the membrane and analyte interaction hinders the forward binding event of the antibody, negatively affecting the sensor performance.^7^ To address these challenges, a number of researchers have explored replacing the conventional membrane with other materials^8,9^ or shifting its focus toward polymer-based channel systems, eliminating the need for membrane materials,^10^ thereby improving the assay sensitivity by 1 order of magnitude.^11^ To replace the membrane, the transport of the fluid and immobilization of biorecognition elements must be reassessed. Fluid can be managed passively, utilizing capillary forces, or actively, necessitating external forces.^12^ Regarding the substrate for immobilization, several polymers such as poly(ethylene terephthalate) (PET), poly(methyl methacrylate) (PMMA), and cyclic olefin copolymer (COC) were used in biosensors due to their low costs^13^ with planar two-dimensional (2D) or three-dimensional (3D) immobilization strategies.^14^ 2D approaches typically employ adsorption or covalent immobilization where the density and amount of the biorecognition molecules are limited to the active sites on the polymer surface itself.^15^ In some instances, the polymers’ hydrophobicity can lead to partial denaturing of proteins so that tethers or spacers need to be employed.^16^ In contrast, 3D immobilization in a polymer matrix such as a hydrogel by encapsulation, copolymerization, electrostatic capture, or covalent linking enables higher immobilization rates within a protective protein surrounding.^17^ Here, the intrinsic swelling behavior of the hydrogel requires an additional washing step to prevent nonspecific signals,^18^ and the overall chemistry involved may be more complex than those for simple adsorptive strategies. To overcome these limitations, recent studies have demonstrated the high efficiency of protein immobilization using polyelectrolyte multilayers (PEMs) as the immobilization matrix.^19^ To accomplish this, polyelectrolytes with opposite charges are alternately deposited onto the desired substrate through dip coating, spraying, spinning, and microfluidics,^20^ demonstrating the high functionality of proteins within such layers such as the immobilization of a polyelectrolyte–protein complex (PPC), where the lysozyme retained all of its enzymatic activity.^21^ Here, we studied and developed a novel, membrane-free LFA concept with 3D streptavidin multilayers as the detection zone and fluorescence nanoparticles as labels. An external pump was employed to control the immunoreaction, while fluorescence microscopy was utilized for POC detection and quantification of NT-proBNP. This biomarker is associated with heart failure (HF),^22^ which stands as the cardiovascular disorder with the highest mortality, morbidity, and healthcare costs.^23^ NT-proBNP is considered the gold standard biomarker, attributed to its longer half-life of 120 min, in contrast to BNP with a half-life of 20 min. This leads to roughly 6 times higher concentrations of NT-proBNP in serum, making it more easily detectable.^24^ With a minimal sample volume of just 15 μL, a rapid detection time of only 20 min, and the ability to integrate a pump and fluorescence detection into a compact and portable support device, this innovative concept successfully addresses the limitations associated with membrane-based lateral flow assays. Additionally, it offers a convenient alternative for finger-prick testing, suitable for both home use and low-resource settings.

## Materials and Methods

The biotinylated capture antibody (polyclonal NT-proBNP sheep-IgG-biotin, cAb), antigen (NT-proBNP (1–76) amid) in buffer or human serum, probe antibody (monoclonal NT-proBNP mouse-IgG), probe antibody-modified fluorescence nanoparticles (Ab-fluorescence NPs), and polystreptavidin (pSA) were provided by Roche Diagnostics GmbH (Mannheim, Germany). Hydrochloric acid (HCl, 0.1 M, 1 M), sodium chloride (NaCl, p.a.), bovine serum albumin (BSA, >96%), poly(diallyldimethylammonium chloride) (PDDA, *M*_w_ 200,000–350,000, 20 wt % in H_2_O), poly(acrylic acid, sodium salt) solution (average *M*_w_ 15,000, 35 wt % in H_2_O), ethylenediaminetetraacetic acid (EDTA, ≥98.5%), sodium hydroxide (NaOH, 1 M), poly(ethylene glycol)-*block*-poly(propylene glycol)-*block*-poly(ethylene glycol) (Synperonic PE/P84), sodium azide, Tween 20 (>97%), and biotinylated Rhodamine 6G (Rh-6G, 0.05 mg·mL^–1^) were supplied from Sigma-Aldrich (www.sigmaaldrich.com). Sucrose was purchased from Serva (www.serva.de). NT-proBNP hs cobas 232 was provided by Roche Diagnostics (Mannheim, Germany).

The LFA consists of the substrate and spacer Melinex329 (175 and 250 μm), which was purchased from Dupont Teijin Films (www.dupontteijinfilms.com), the cover foil Hostaphan RN 100 was purchased from Mitsubishi Polyester Film (www.m-petfilm.de), and the double-sided adhesive tape was supplied from Henkel-Adhesives (www.henkel-adhesives.com).

HEPES-buffered saline (HBS) consisted of 50 mM HEPES, 150 mM NaCl, 3 mM EDTA, and 0.05% (w/v) Tween 20 and was adjusted to pH 7.4. HEPES dispensing buffer was prepared with 50 mM HEPES, 1% (w/v) albumin, 1% (w/v) sucrose, 0.15% (w/v) synperonic PE/P84, and 0.024% (w/v) sodium azide and was adjusted to pH 7.4.

For particle characterization, Zetasizer Ultra Pro (www.malvernpanalytical.com) was used. All drying procedures were done with the drying cabinet at 50 °C (FED 400 E2, www.binder-world.com). For plasma treatment, a plasma oven (www.gs-technologie.de) was used.

### Pretreatment of Substrate and Blocking of Nonspecific Adsorption

First, the substrate (MELINEX329 175 μm) was pretreated with oxygen plasma for 2 min and 100 W to increase the hydrophilicity and thus enhance the polyelectrolyte multilayer attachment. For blocking nonspecific adsorption, a thin poly(acrylic acid) (PAA) layer was used. Therefore, the base of the microfluidic sensor was dip-coated with PAA (0.2% (w/v) and 150 mM NaCl, pH 7.4) for 60 s, washed with distilled water, and dried at 50 °C and then utilized for polyelectrolyte streptavidin multilayer deposition.

### Polyelectrolyte–Protein Complex (PPC) Fabrication

For the fabrication of the PPCs, a modified procedure of vander Straeten et al. is utilized.^21^ PPCs were fabricated by mixing poly(diallyldimethylammonium chloride) (PDDA) with polystreptavidin (pSA). First, a PDDA solution was prepared in 150 mM NaCl, and polystreptavidin was dissolved in water to reach an end concentration of 0.5% (w/v) and 10 mg·mL^–1^, respectively, and pH was adjusted to 7.4. Then, 500 μL of PDDA was mixed with 500 μL of pSA to generate the functionalized particles. The PPCs were stored at 4 °C until further use.

### Fluidic-Assisted PPC Multilayer Lane Assembly and Antibody Dispensing

PPC multilayer lanes were deposited onto the base of the microfluidic using a fluidic immobilization channel. To assemble the polyelectrolyte multilayer (PEM), the PPC solution and poly(acrylic acid) (PAA) solution were alternately deposited onto the base by filling the immobilization channel solely by capillary forces. PAA was dissolved in 150 mM NaCl to a concentration of 0.5% (w/v) and was adjusted to pH 4.55. The fluidic deposition channel with double-sided adhesive tape was bonded onto the PET substrate and used as a shaping lane mold. By using a stop-flow approach, the channel was filled with the PPC solution and deposited for 60 s. After the deposition time, the capillary was emptied and filled with the PAA solution for 60 s. This alternate coating process was repeated for a given number of cycles for the preparation of the PEMs. Afterward, the channel mold was relieved from the substrate, and the (PPC/PAA) multilayer was dried at 50 °C for 5 min. In the next step, 1.5 μL of each antibody was dispensed onto the PET substrate and dried at 40 °C for 3 min. The substrate with the streptavidin lane and probe antibodies then adhered to the spacer and cover foil to finish the membrane-free LFA. The ready-to-use LFAs were stored at 4 °C until further use. Details of the fabrication process are provided in the Supporting Information.

### Biofunctionality of PPCs

To investigate the biofunctionality of polystreptavidin within the prepared PPCs in the multilayer, the streptavidin multilayer was tested with the biotinylated Rhodamine 6G (Rh-6G, λ_ex_ = 525 nm, λ_em_ = 548 nm). Rh-6G was prepared in a 0.05 mg·mL^–1^ stock solution with distilled water. Twenty microliters of the Rh-6G solution was injected into the LFA and actively transported over the streptavidin multilayer for 5 min. The unbound solution was removed and the streptavidin multilayer was then analyzed with fluorescence microscopy.

### Assay Equipment

The customized fluid control equipment and fluorescence microscopy were provided by Roche Diagnostics GmbH (Mannheim, Germany) (Figure S2). Details of the fluid control and fluorescence microscopy are provided in the Supporting Information.

### Performance of the Bioassay

A sandwich immunoassay with both buffer and plasma samples was carried out to investigate the assay performance of the bioassay. The analyte target was the N-terminal prohormone of brain natriuretic peptide (NT-proBNP), which is a biomarker for heart failure. NT-proBNP biomarker samples were prepared in the HBS buffer and human plasma through the dilution of a stock solution to produce concentrations of 7.5, 15, 30, 60, 125, 250, 500, 1000, 2000, 4000, 6000, and 9000 pg·mL^–1^ for analysis. The immunoassay was performed at room temperature. For the calculation of the limit of detection (LOD), the logistic fit parameter for the lower curve asymptote *A* and the standard deviation of the blank SD (blank)
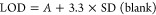
1

## Results and Discussion

**Figure 1:**
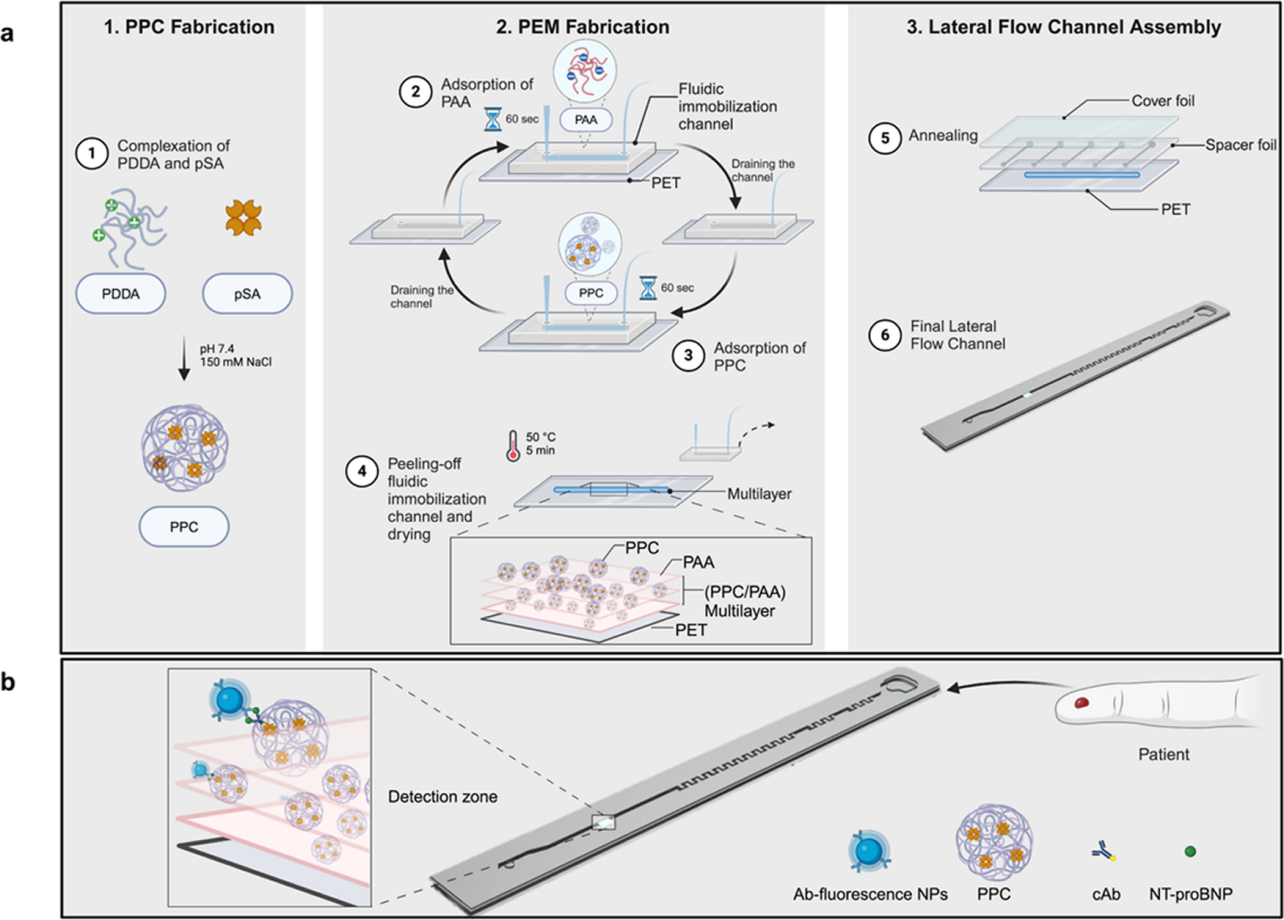
Schematic (not to scale) illustration of the membrane-free POC device. Fabrication of the detection zone consists of two steps (a): fabrication of the protein–polyelectrolyte complex (PPC) by the complexation of pSA and PDDA at pH 7.5, 150 mM NaCl (1) and subsequently layer-by-layer deposition of poly acrylic acid (PAA) and PPCs on the PET substrate of the sensor (2–4). The sensor was then annealed with the spacer and cover foil (5). The final lateral flow channel (6). Schematic (not to scale) illustration of the sensing principle (b). Ab-fluorescence NPs: probe antibody-modified fluorescence nanoparticles; cAb: biotinylated capture antibody. Adapted from “PDMS Microfluidic Chip Fabrication” by BioRender.com (2023). Retrieved from https://app.biorender.com/biorender-templates.

The main goal of this study was the design of a POC device that does not require membranes and can hence function with very small sample volumes (finger-prick sampling) while maintaining the simplicity of use and sensitivity afforded by the current LFAs (Figure 1). The detection is accomplished using fluorescent immunobeads as described previously by Lutz et al.^25^ The immobilization of the biorecognition elements in a test line was achieved by designing protein–polyelectrolyte complexes (PPCs) consisting of polystreptavidin-poly(diallyldimethylammonium chloride) composite microparticles, which were then integrated into a charged polymer multilayer. Fluid transport and the overall assay protocol were finally optimized for the sensitive detection of NT-proBNP (Figure S3).

### Characterization of PPCs

PPCs were studied to develop a novel, sensitive POC immunoassay. More specifically, we utilized the cationic poly(diallyldimethylammonium chloride) (PDDA) and the negatively charged polystreptavidin (pSA) for the synthesis of biofunctional microparticles. The characteristics of the protein–polyelectrolyte complex, especially the size and the charge of the complex depend on the nature and intensity of the polyelectrolyte–protein interactions and on the environment.^26^ Hence, we explored the impact of the polyelectrolyte-to-protein ratio and the pH on the complexation process. The latter has a major effect on the ionization of the materials used. Whereas PDDA, as a strong cationic polyelectrolyte, has a positive charge over a wide pH range (pH 2–13) and is stable to pH shifts, the amphoteric polystreptavidin with the isoelectric point (IEP) of 5–6^27^ is notably influenced by the pH of its environment. To ensure the complexation of PDDA and pSA, both materials were mixed in different ratios at pH 7.4 and 150 mM NaCl (Figure S4). This pH level is slightly higher than pSA’s isoelectric point (IEP), ensuring the overall negative charge of pSA, which in turn facilitates the electrostatic complexation with PDDA. Considering the physiological environment of the envisioned assay, a pH of 7.4 and ionic strength of 150 mM NaCl were hence kept constant in all subsequent experiments. Directly after the mixing process, highly turbid samples were obtained immediately. DLS analysis and zeta potential measurements proved the complexation process.^28^ The size and polydispersity index (PdI) of the resulting composite particles were dependent on the PDDA:pSA ratio (Figure S4a). In particular, an excess of pSA led to the attainment of maximum size with a minimal polydispersity index (PdI), indicative of an intricate mesh-like network wherein streptavidin serves as a bridge connecting two or more PDDA chains. In this context, there are segments on the PDDA chain without attached streptavidin. These unbound portions generate electrostatic repulsion, effectively preventing aggregation and complete collapse.^29^ However, an excess of PDDA led to the formation of small particles characterized by relatively high PdI values. This suggested that the limited amount of streptavidin is bound in a densely compacted manner, effectively covered and regulated by PDDA. The high PdI can be explained by the fact that some parts of the PDDA stick out at the surface, like a core corona structure.^30^ Regarding the zeta potential of the resulting composite material, it is evident that it naturally becomes increasingly positive as the PDDA:pSA ratio rises (Figure S4b). In this context, it is likely that PDDA completely envelops the particles. All of these were used in subsequent studies of the novel LFA concept.

### Formation of the 3D Test Line and Assessing the Biofunctionality of the Embedded PPCs

On the PET substrate, the 3D test line was generated in a layer-by-layer approach of the composite PPCs and poly(acrylic acid) (PAA). The PET substrate underwent oxygen plasma treatment to enhance its hydrophilic properties, a crucial step for facilitating the adhesion of bioactive coatings. Specifically, the plasma treatment induces increased surface roughness, thereby expanding the number of available attachment sites.^31^ In general, a simple dip coating process in which the substrate is alternately dipped into the PE solutions is used to generate multilayers. The average dip coating time of each layer is 15 min in order to guarantee the PAA adsorption and charge overcompensation in the multilayer assembly.^20^ Moreover, a washing step is necessary between each layer deposition to wash away unbound protein or PAA, resulting in a time-consuming and material-wasting procedure. Here, for the layer-by-layer process, a fluidic immobilization channel was used as a lane-shaping mold that was attached to the PET foil.^32^ First, the positively charged PPCs were deposited onto the negatively charged PET substrate by capillary-driven filling of the channel. Subsequently, the anionic PAA was applied through the same process. By alternating these two steps, multilayers of 2–12 layers were created. It was determined that the deposition time of PAA and PPCs affected the multilayer performance. The deposition is hereby solely dependent on the Brownian diffusion to the bottom layer of the multilayer. Through the utilization of a material-efficient fluidic-assisted stop-flow multilayer deposition method, we achieved a more economical and time-efficient procedure, eliminating the need for a washing step.^33,34^ Due to the geometry of the immobilization channel, the (PPC/PAA) multilayer assembly was completed within only 8 min. To assess the biofunctionality of pSA when inside the PPC/PAA multilayer lane, the binding efficiency of a biotinylated Rhodamine 6G (Rh-6G, λ_ex_ = 525 nm, λ_em_ = 548 nm) to pSA within the multilayer was investigated. Subsequently, the immobilization channel was removed and the actual LFA was assembled (Figure 1). The 20 μL Rh-6G solution was transported to the test line and incubated for 10 min. After that, the channel was emptied to remove unbound dye molecules and the fluorescence was quantified using fluorescence microscopy and ImageJ. The binding of the dye by streptavidin within the multilayer was investigated depending on the PDDA:pSA ratio (Figure S5). The lowest fluorescence signals were found at low and high PDDA concentrations. At low PDDA concentrations, large PPCs with low positive charges were generated, which represented a loose network. It is assumed that this may encourage streptavidin leaching, as it is insufficiently bound by PDDA. In addition, the particles only show a slightly positive charge (Figure S4b), which results in reduced electrostatic interactions with PAA during the multilayer deposition process. Conversely, an excess of PDDA can lead to competition between PDDA and PPCs for the available electrostatic binding sites with PAA during the multilayer formation, resulting in a reduced amount of streptavidin within the multilayer. Overall, the maximum fluorescence intensity was found at the PDDA:pSA ratios of 0.5 and 1, suggesting that the highest amount of streptavidin could be securely immobilized within the multilayer.

Due to economic reasons, the PDDA:pSA ratio of 0.5 was used for further experiments.

### Development and Optimization of the Novel LFA Concept

**Figure 2:**
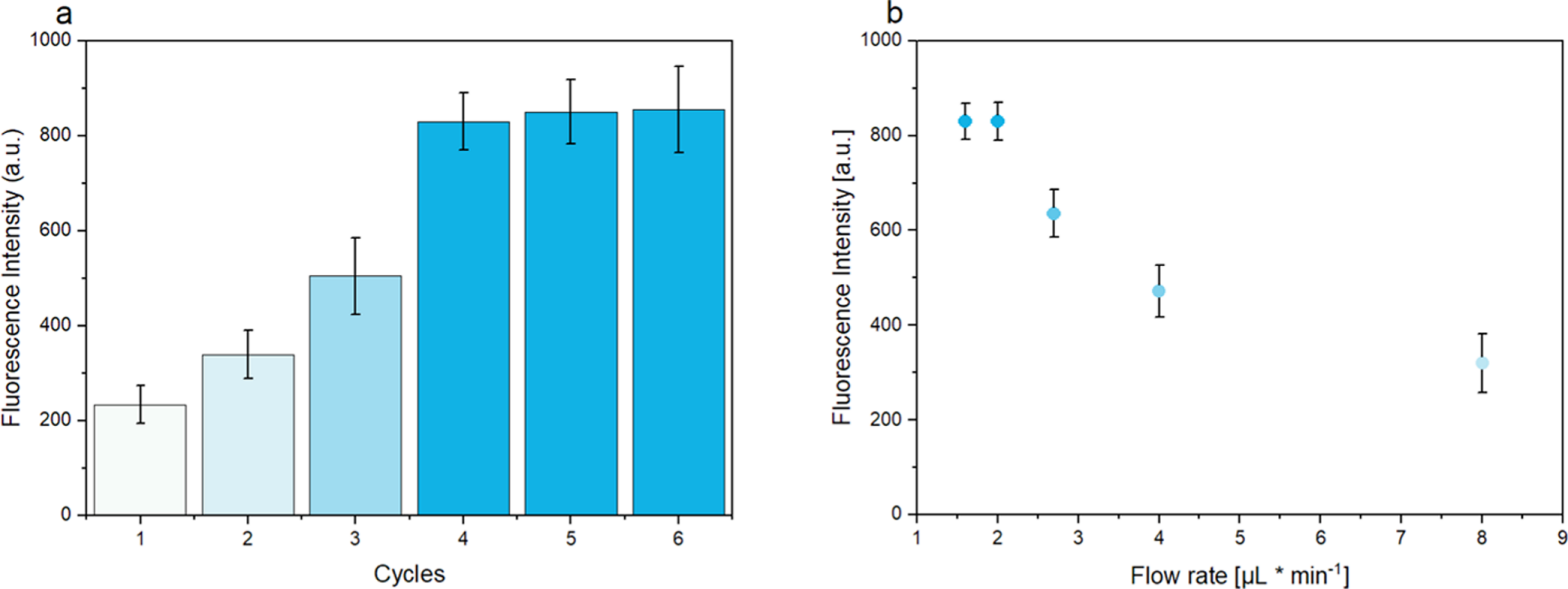
Plot of fluorescence intensity of the bioassay using a constant antigen concentration of 1 ng·mL^–1^ in buffer against the number of layer-by-layer cycles (a). Plot of the fluorescence intensity of the bioassay using a constant antigen concentration of 1 ng·mL^–1^ in buffer against the flow rate (b). Error bars represent mean values ±1σ and were calculated based on three parallel measurements on three different LFAs (*n* = 3).

After the initial proof-of-principle multilayer formation, we further optimized its performance in an antibody-based sandwich LFA. First, the number of (PPC/PAA)_*x*_ layers was investigated using a sandwich assay of biotinylated capture antibodies (cAb), fluorescent reporter antibodies (Ab-fluorescence NPs), and NT-proBNP as an analyte (Figure 2a). It was found that fluorescence signals increased significantly up to the first (PPC/PAA)_4_ multilayers, which suggests that the immobilized pSA concentration increased initially with the number of layers.^35^ Additional layers did not lead to a further signal increase. Furthermore, the need for the composite PPC in contrast to mere polystreptavidin in the multilayer buildup was studied (Figure S6). It was found that even a high concentration of pSA (15 mg·mL^–1^) yielded only low fluorescence signals. It is assumed that the strong and homogeneous positive charge of the PPCs leads to a stronger interaction with the PAA polyelectrolyte layers, potentially leading to an increased concentration of streptavidin within the multilayer. These findings were in accordance with the work of vander Straeten et al., who showed higher immobilization rate of the complexed protein than the pure protein itself in the multilayer.^21^ Second, the impact of the flow rate on the assay was assessed. As the flow rate has an influence on the immunoreaction and on the rehydration characteristics of dried reagents, we initially dried biotinylated cAbs and Ab-fluorescence NPs on the PET substrate. The 20 μL sample volume was transported with a flow rate of 8, 4, 2.7, 2, and 1.6 μL·min^–1^. It was found that high flow rates resulted in low fluorescence signals (Figure 2b) most likely due to less efficient capturing of the immune sandwich and insufficient rehydration of the dried reagents. In contrast, at flow rates of ≤2 μL·min^–1^, high fluorescence signals were obtained, reaching a plateau. Therefore, a flow rate of 2 μL·min^–1^ was chosen for further experiments. Decreasing the nonspecific binding of sample matrix components onto the PET substrate was studied using BSA as a commonly used blocking agent in bioassays^36^ and PAA, a highly hydrophilic polyelectrolyte (Figure S7).^37^ First, the channel was filled with 40 μL of the blocking solution, containing either BSA or PAA and then incubated for 10 min. After draining the channel, the LFAs were dried in an oven at 50 °C. Subsequently, an immunoassay was performed by applying 20 μL of the sample to the assay. High nonspecific signals were found when no blocking was performed and when BSA acted as a blocking agent. In the case of no blocking, it can be assumed that plasma proteins deposit on the channel surface, then further promoting the nonspecific binding of the fluorescence nanoparticles. Similarly, when the channel surface is blocked with BSA, it is assumed that BSA molecules will adsorb on the channel surface and interact with the fluorescence latex beads.

In contrast, when the hydrophilic PAA is used as a blocking agent, nonspecific signals could be significantly decreased. Therefore, the channel surface was treated with PAA for subsequent experiments. Eventually, the concentration of the cAb and Ab-fluorescence NPs was optimized with respect to the strongest fluorescence intensity for a constant analyte concentration (Figure S8). For the maximum fluorescence signal, the cAb was prepared at a concentration of 2.5 μg·mL^–1^ and the Ab-fluorescence NPs were prepared at a concentration of 2% (w/v) in the HEPES dispensing buffer.

### Investigating Storage Stability of the Novel LFA Approach

**Figure 3:**
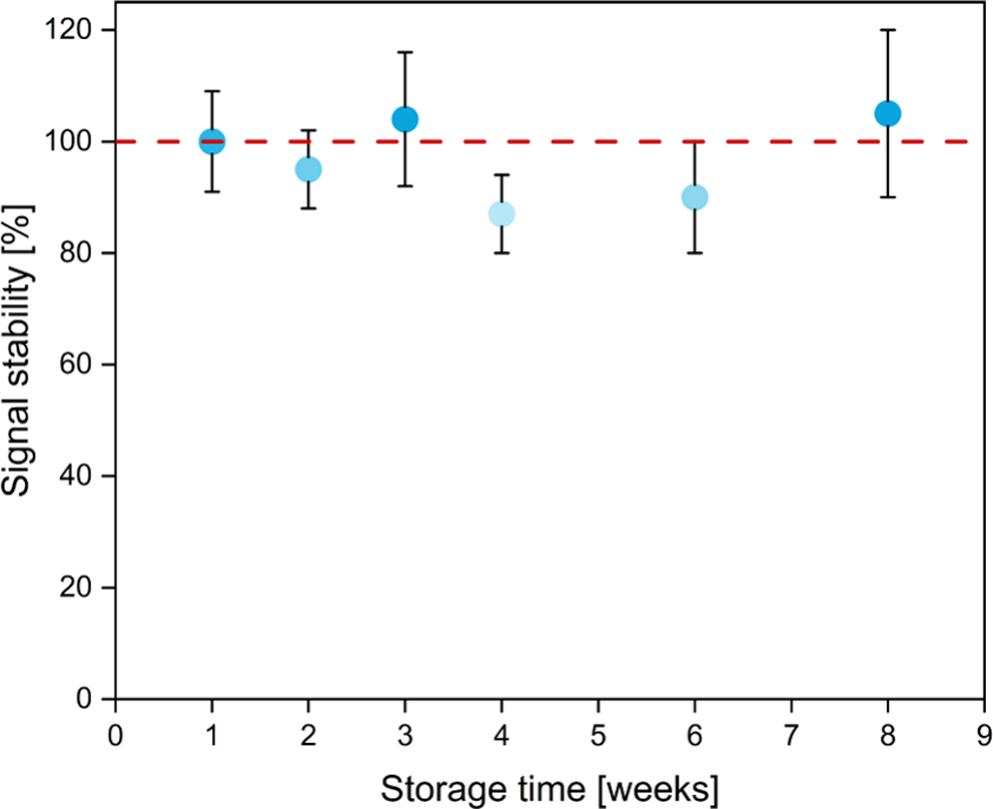
Stability of the fluorescence signal of the bioassay using a constant antigen concentration of 1 ng·mL^–1^ over 8 weeks. The LFA was stored in an airtight capsule with a drying agent at 4 °C. Error bars represent mean values ±1σ and were calculated based on three parallel measurements on three different LFAs (*n* = 3).

The overall stability of the assembled LFA was investigated. While the antibodies and polystyrene Ab-fluorescence NPs are known to be stable when stored in the dry stage through a fleece-based system,^2^ their storage within a nonfleece POC immunoassay is not yet known. Antibodies have been successfully stored in dry stage on a plastic support within a sugar-based matrix and showed stable functionality over long time period.^38^ Thus, the capture antibodies and polystyrene Ab-fluorescence NPs were dried in a sucrose matrix, supporting the stability maintenance of both reagents in the dried state. Over a period of 8 weeks, no loss in activity could be observed (Figure 3). In addition, we assume that the complexation of streptavidin and the incorporation of the PPCs in a multilayer also contributed to stable and reproducible signal values. This effect is linked to the additional hydrated state of the assembly, having a protective effect on the immobilized protein.^21^ The signal’s overall variability can be attributed to variations in multilayer formation, as the manual fabrication process may lead to differences in multilayer thickness, impacting the immobilized streptavidin concentration. We assume that an automated process for building up the multilayer would result in a more constant multilayer thickness, potentially reducing signal variability. The elevated relative errors, approximately 20%, could be justified by handling inaccuracies and the limited number of replicates in the handmade LFAs utilized.

### Detection of the NT-proBNP Quantification in Buffer and Serum with a Novel LFA System

**Figure 4:**
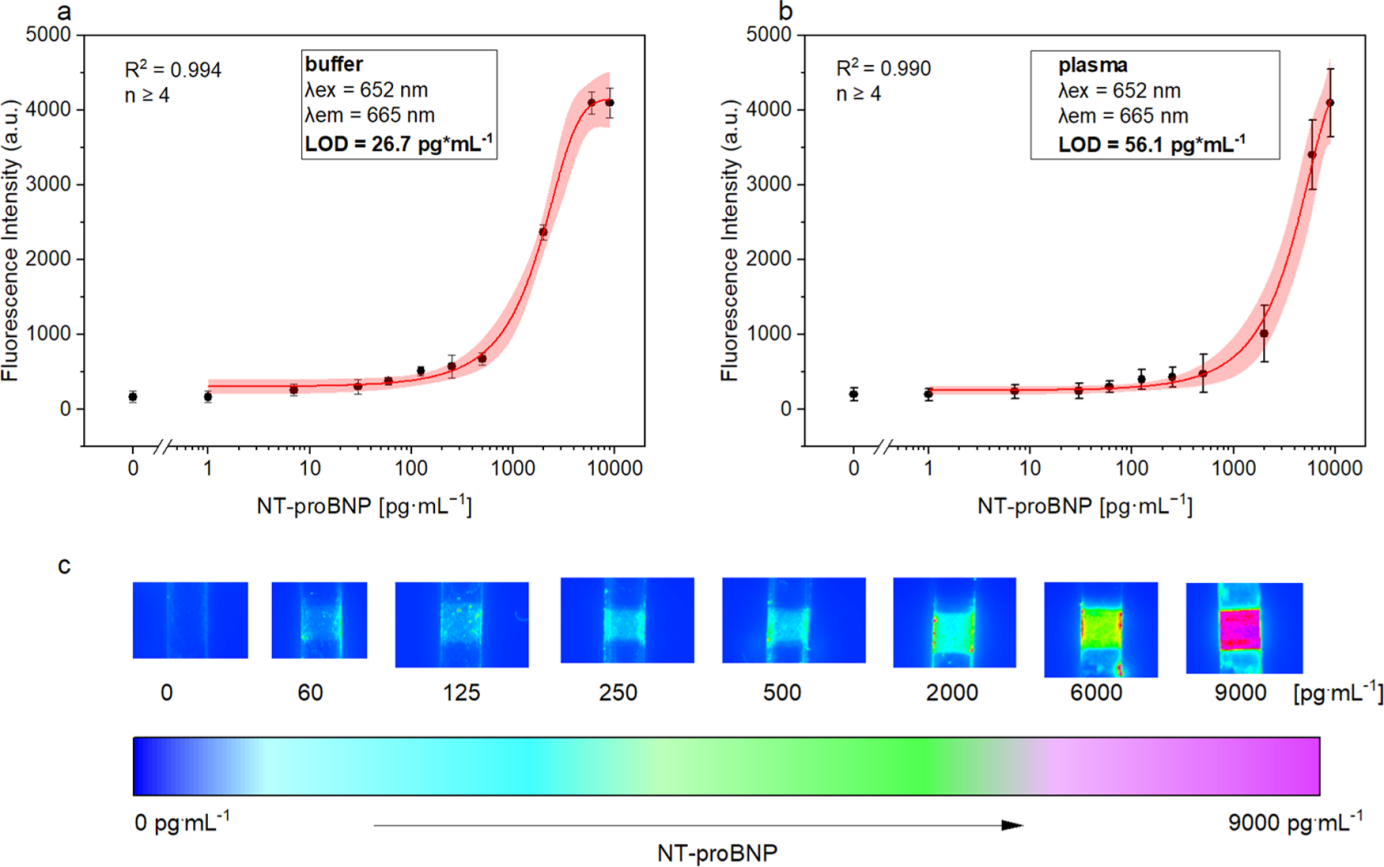
Plot of fluorescence intensity against the logarithm of antigen concentration in a spiked HBS buffer (a) and in spiked human serum samples (b) with the logistic fit (red line), confidence interval 95% (shaded red curve), and the corresponding parameters. Fluorescence images of the detection zone illustrating the antigen concentration (c). Standard deviation was calculated based on six parallel measurements on six different LFAs, while outliers were removed after the *Q*-test (confidence interval 95%). Error bars represent mean values ±1σ (*n* ≥ 4).

To demonstrate the applicability of the LFA in a complex biological matrix, the bioassay was performed with both buffer and human serum samples that were spiked with different amounts of NT-proBNP (Figure 4). In the plot of the fluorescence intensities against logarithm NT-proBNP concentration, the typical sigmoidal curve for sandwich immunoassays is obtained. Based on the logistic fit for the assay with spiked buffer, a LOD of 27 pg·mL^–1^ was calculated. The assay with human plasma showed a LOD of 56 pg·mL^–1^. The slight decrease of the LOD is attributed to the higher blank value due to the higher background signal originating from the nonspecific binding of the nanoparticles and autofluorescence of the plasma.^39^ Additionally, we observed irreversible adsorption of the sample and antibodies on the channel sides when the sensor was not bonded appropriately due to manual inaccuracies in the fabrication process. This led to increased variability in the signal, particularly noticeable when the analyte was present in low concentrations. This is also reflected by the mean coefficient of variation (CV) of the assay with the buffer and plasma, which was calculated to be 14 and 19%, respectively. The dynamic range of both assays is similar and extends as expected for an immunoassay over nearly 2 orders of magnitude. Most importantly, the new LFA compared very well to the current commercial LFA, the optical NT-proBNP cardiac POC-system (hs cobas 232) from Roche Diagnostics, which showed a LOD of 60 pg·mL^–1^ and a dynamic range of 60–9000 pg·mL^–1^.^40^ A NT-proBNP value of greater than 100 pg·mL^–1^ is abnormal in patients and is used to assess the severity of heart failure. Thus, this study shows that the membrane-free LFA can be used for the diagnosis and prognosis of heart failure and, furthermore, that no membrane is needed to achieve the same assay performance as the hs cobas 232 NT-proBNP (Table 1).In addition, only 20 μL of plasma is needed for the new LFA, which suggests that it can, in fact, be used as a POC assay with samples derived from a finger-prick sampling.

**Table 1:** Determination of NT-proBNP^a^

	CV	
NT-proBNP amount spiked (pg·mL^–1^)	buffer (%)	plasma (%)
60	18	22
125	16	21
250	19	19
500	13	22
2000	11	21
6000	8	14
9000	9	11

a 20 μL of the sample volume was used for each test. Samples were run in sextuplicates, and the mean was reported (*n* = 6).

## Conclusions

In summary, we introduce an innovative membrane-free and pump-driven fluorescence lateral flow assay (LFA) ready to meet the demands of one-step POC applications. By utilizing composite polystreptavidin–polyelectrolyte multilayers as the detection zone and adopting a dry-storage approach for antibodies and fluorescence labels in our platform, the test not only simplifies assembly and reduces production costs but also achieves high sensitivity. Furthermore, our platform overcomes the typical limitations of traditional membrane-based LFAs, such as variations in membrane fabrication, limited control over immunoreactions, and the need for large sample volumes. Healthcare of the future will require reliable tests that can be conducted without the need for medical personnel while still offering the quantitative and highly sensitive features of standard laboratory tests. The membrane-based LFA concept currently dominating the market can provide rapid answers with reasonable limits of detection but without reliable quantification and not at often-needed low analyte concentrations. The developed test homes in on the need for less sample volume and on a quantitative, sensitive, and reliable measurement at the POC. The design allows the usage of a 7.5-fold smaller volume of sample when compared to the conventional LFA format to achieve the same assay performance without sampling through the vein and the need for intricate washing procedures. This allows straightforward testing without trained personnel and significantly improves patient comfort, especially when a finger-prick sample volume is sufficient. Active sample transport delivers the precise control necessary for immunoreaction and, when combined with a fluorescence imaging system, enables highly sensitive detection, even if instrument-free visual detection is no longer possible. With the goal of increasing the simplification of the developed LFA, future improvements may include the introduction of a hand-held device that combines a fluorescence detection camera, a pump system, and relevant software/apps for automated analysis that can be easily installed into POCT devices. With the ongoing trend of miniaturization and the rise of the Internet of Medical Things (IoMTs), the hardware has become cheap and allows straightforward testing and decision-making at home and in resource-limited settings.^41^ Moreover, we will focus on expanding the variety of analytical targets that can be detected and quantified using the developed multilayer platform. Multiplexing can be readily achieved by establishing separate channels where precise control of the sample is maintained for each analyte. In addition, future work will seek to implement a blood–plasma separation mechanism to promote the practical applications in low-resource areas. Hence, this work may provide a new strategy to produce sensitive quantitation of HF biomarkers. We, therefore, demonstrated that our membrane-free platform provides the quantitative and highly sensitive characteristics for the HF biomarker NT-proBNP.

## Supporting Information Available

Additional details on fabrication; buildup of the LFA in the side view and in the top view; photographs of the assay equipment; optimization of the PDDA:PSA ratio in the complexation process; optimization of cAb and dAb (PDF)
