# Supplementary material for: Membrane-Free Lateral Flow Assay with the Active Control of Fluid Transport for Ultrasensitive Cardiac Biomarker Detection
Source: Anal Chem. 2024 Apr 25;96(18):7014–21. doi: 10.1021/acs.analchem.4c00142 (PMC11079857; doi:10.1021/acs.analchem.4c00142)
Supplement: Supplementary file 1 — ac4c00142_si_001.pdf [file ac4c00142_si_001.pdf]

Supporting Information for

A membrane-free lateral flow assay with active control of fluid transport for ultrasensitive cardiac biomarker detection

Dan Strohmaier-Nguyen, Carina Horn[b], Antje J. Baeumner[a] [1]

[a] *University of Regensburg, Institute of Analytical Chemistry, Chemo- and Biosensors, 93043 Regensburg*

[b] *Roche Diagnostics, 68305 Mannheim, Germany*

Table of contents:

- Text S1. Material and Methods
- Figure S1-S8

[1] Corresponding author.
E-mail address: antje.baeumner@ur.de (A.J. Baeumner).

Text S1. Material and Methods

Lateral flow immunoassay materials and fabrication

LFAs were built up from four components: 1) a PET foil as substrate, 2) a spacer with laser cut capillaries, 3) a cover foil with sample port and outlet port and 4) pressure sensitive double sided adhesive tape. The spacer (MELINEX®329 250µm) was coated with double sided adhesive tape on both sides and then capillaries (dimension: 90 mm x 1.5 mm x 0.28 mm) were cut with the laser MicroLine 6000 P (www.lpkf.com) at a frequency of 30 kHz and 3 W. The second laser-cutting step was used to generate the sample inlet and outlet in the cover foil. The spacer and cover foil were then stick together as depicted in the ESM (Fig. S1) and finally joined on top of the base, orthogonal to the streptavidin multilayer, to get the ready to use microfluidic sensor. The fluidic immobilization channel (dimension: 90 mm x 1.5 mm x 0.28 mm) for the deposition of the streptavidin multilayer was similarly prepared with the exception that it only consists of the spacer and the cover foil

Principle of the LFA

Upon adding the sample, the dried antibodies dissolve, initiating the immunoreaction. The pump regulates the sample flow, directing it through the mixing area, thereby enhancing the diffusion-based formation of the immunocomplex. The immunocomplex, owing to robust biotin-streptavidin binding, gets captured in the detection zone, allowing analysis of the fluorescence signal using a fluorescence microscope.

Assay equipment

The fluid control consisted of a metal housing that houses 1) a LFA mold, 2) an Arduino Due board, 3) and a miniaturized vacuum pump. The fluid control system was linked to a computer via USB and managed using an Arduino code. To initiate the immunoassay fhe fluid control was mounted on top of the outlet port of the LFA.

All fluorescence images were acquired using a fluorescence microscope, LINOS lens (www.excelitas.com), HTC camera with a Sony CCD sensor ICX285AL (www.sony.com), XENON XBO R 100W/45 OFR lamp (www.osram.com), 633nm excitation filter and 685nm detection filter (www.semrock.com). The fluorescence images were taken with an exposure time of 25 ms. Image processing and data analyses were carried out with ImageJ and Origin 2021.

Supporting Figures:

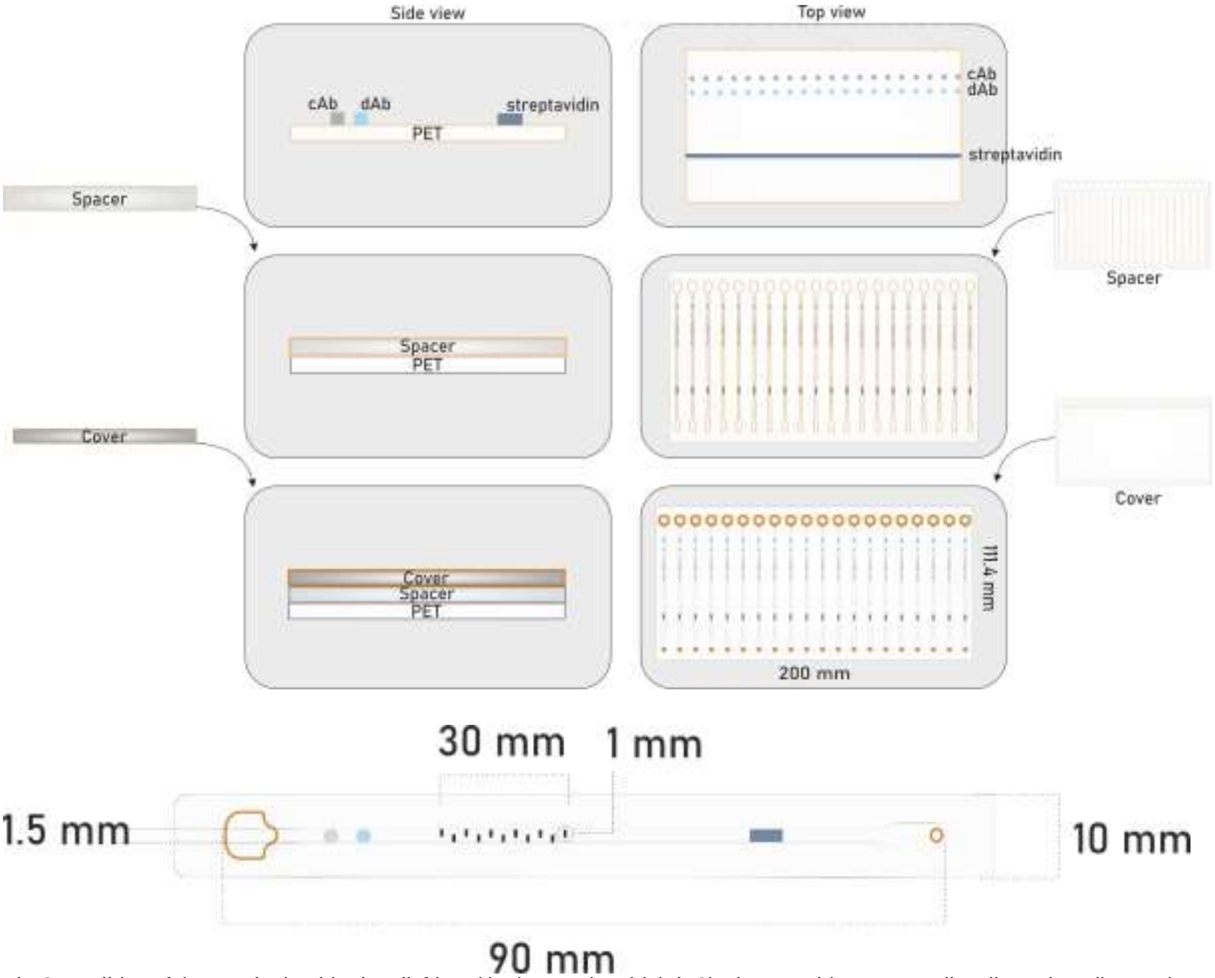

Fig. S1: Buildup of the LFA in the side view (left) and in the top view (right). Single LFA with corresponding dimensions (bottom). cAb – capture antibody and dAb – detection antibody.

a) b)

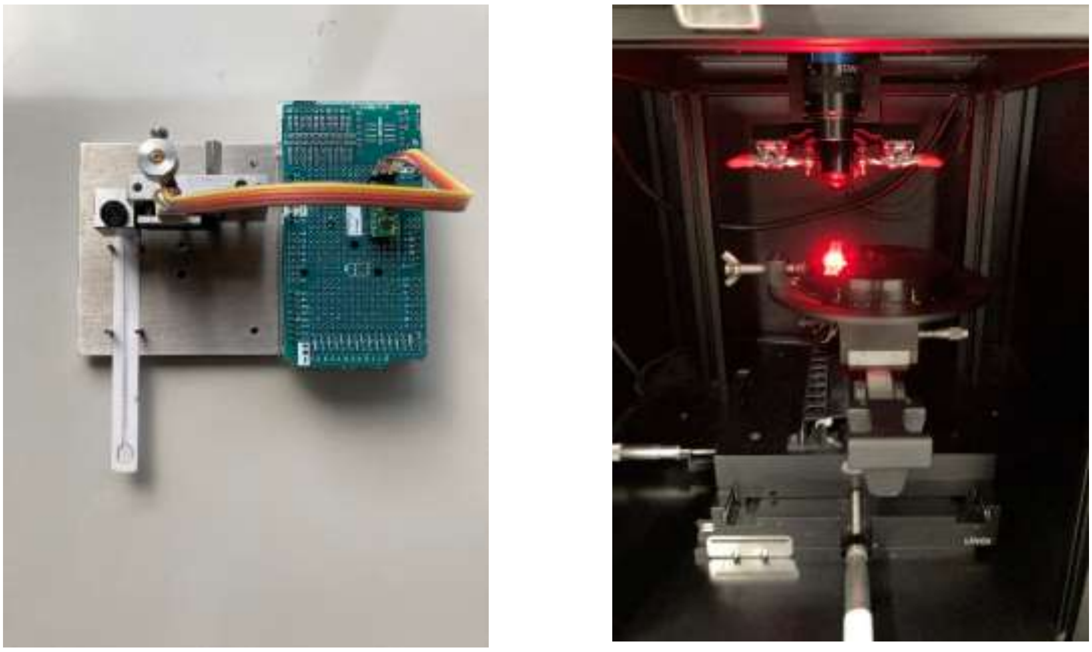

Fig. S2: Photographs of the assay equipment. Fluid control system (a) and fluorescence microscopy (b).

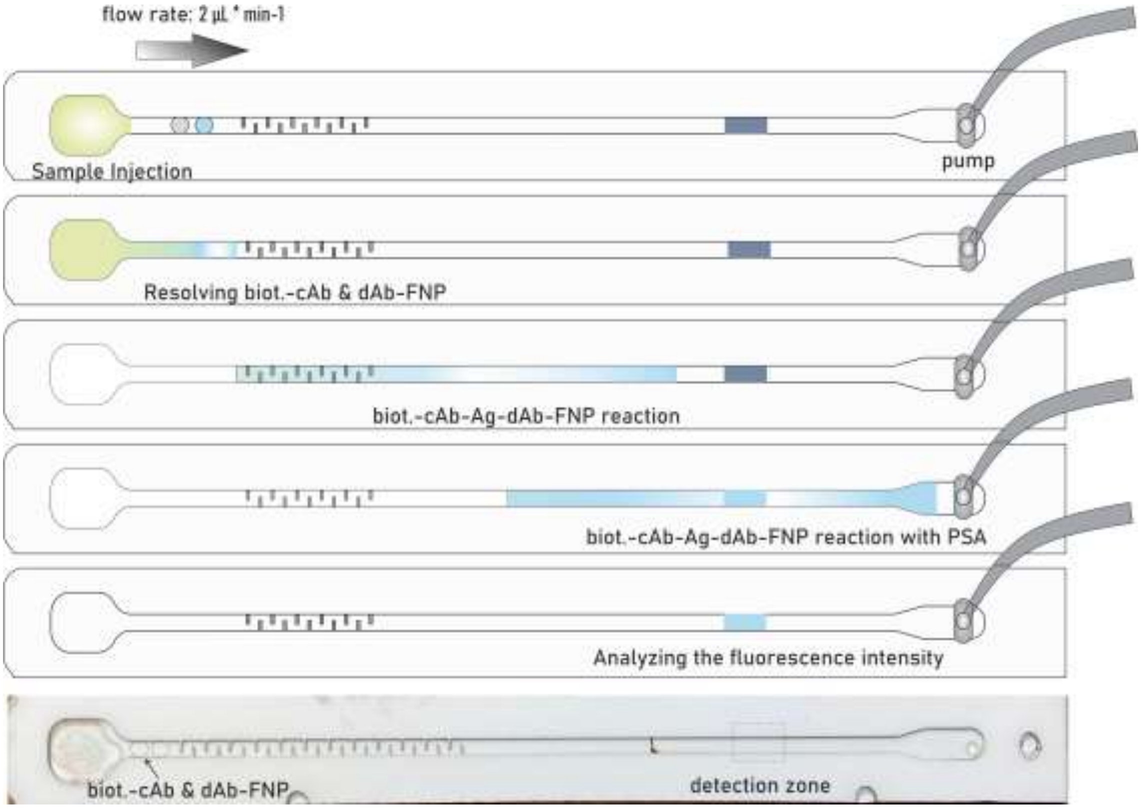

Fig. S3: Schematic (not to scale) illustration of the active sample transport by the pump and process of the immunoreaction.

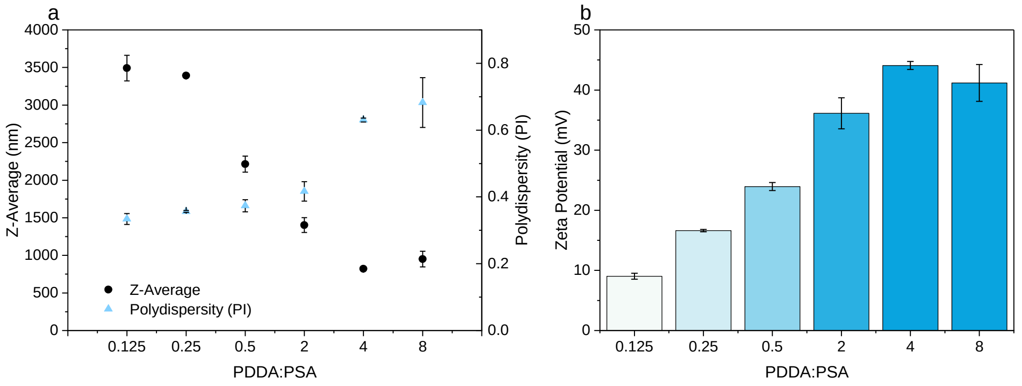

Fig. S4: Characterization of the PDDA:PSA particles at pH 7.4 and 150 mM NaCl and the effect of the PDDA:PSA ratio on the size (a) and on the zeta potential of the PDDA:PSA complex (b). Error bars represent mean values ±1σ. (n = 3)

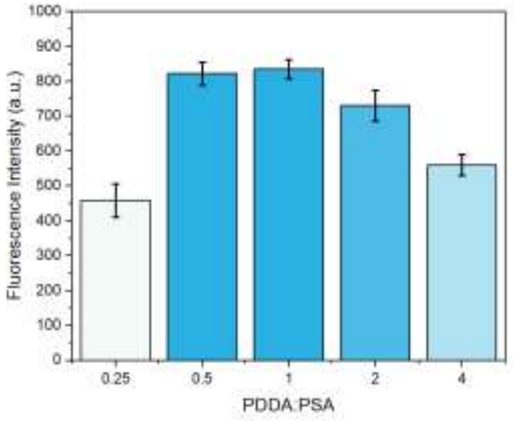

Fig. S5: Optimization of PDDA:PSA ratio in the complexation process. Plot of fluorescence intensity of a constant Rhodamine 6G concentration (0.05 mg*mL^{-1}) against the PDDA:PSA ratio. Error bars represent mean values ±1σ and were calculated based on three parallel measurements (n = 3)

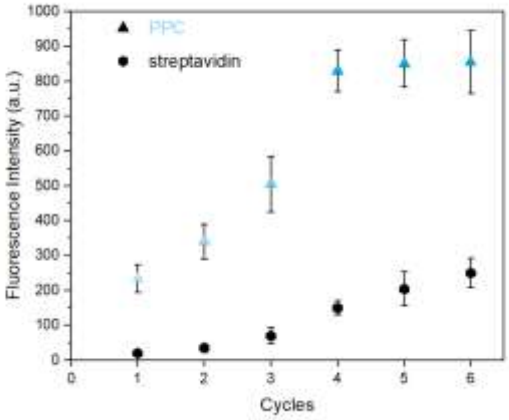

Fig. S6: Plot of the fluorescence intensity of a constant Rhodamine 6G concentration (0.05 mg·mL^{-1}) against the number of layer-by-layer cycles of $(PPC/PAA)_x$ and (streptavidin (15 mg·mL^{-1})/$PAA)_x$. Error bars represent mean values ±1σ and were calculated based on three parallel measurements on three different LFAs (n = 3)

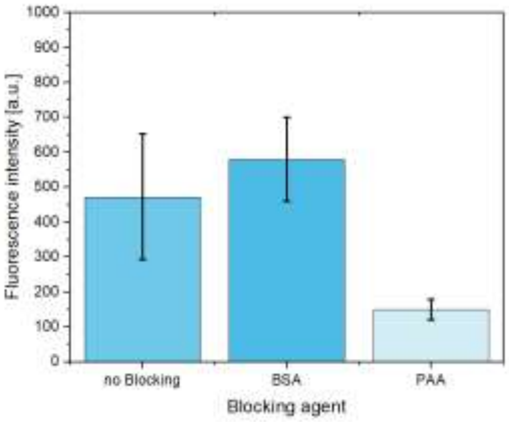

Fig. S7: Plot of fluorescence intensity of the bioassay using a constant antigen concentration of 1 ng·mL^{-1} in buffer against different blocking agents. Error bars represent mean values ±1σ (n = 3)

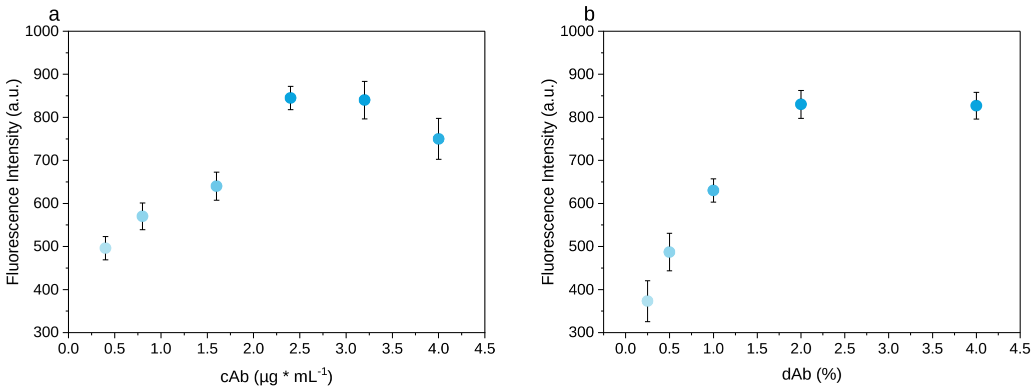

Fig. S8: Optimization of cAb and dAb. Plot of the fluorescence intensity of the bioassay using a constant antigen concentration of 1 ng·mL^{-1} in buffer against the cAb concentration used in the bioassay (a). Plot of the fluorescence intensity of the bioassay using a constant antigen concentration of 1 ng·mL^{-1} in buffer against the dAb concentration used in the bioassay (b). Error bars represent mean values ±1σ (n = 3)
